# Assessing physical activity in daily life, exercise, and sedentary behavior among Japanese moving to westernized environment: a cross-sectional study of Japanese migrants at an urban primary care center in Pittsburgh

**DOI:** 10.1186/1447-056X-13-3

**Published:** 2014-01-14

**Authors:** Nobutaka Hirooka, Teiichi Takedai, Frank D’Amico

**Affiliations:** 1 Department of General Medicine, Japan National Defense Medical College, 3-2 Namiki, Tokorozawa, Saitama, Japan; 2 Shadyside Family Medicine Residency, University of Pittsburgh Medical Center, 5215 Centre Avenue, Pittsburgh, PA, USA; 3 Faculty Development Fellowship, University of Pittsburgh Family Medicine, 815 Freeport Road, Pittsburgh, PA, USA

**Keywords:** Physical activity, Exercise, Sedentary behavior, Japanese in USA, Noncommunicable diseases

## Abstract

**Background:**

Noncommunicable chronic diseases (NCDs) are global public health issues. Physical activity, exercise and sedentary behavior are important lifestyle factors to determine risk of NCDs. Immigrant studies have shown higher risk of developing NCDs among immigrants. Less physical activity among Japanese immigrants to westernized environment was also documented. However, little is known about detailed physical activity, exercise and sedentary behavior among Japanese residing in westernized environment. This cross-sectional study was conducted to analyze physical activity in daily life, exercise, and sedentary behavior among Japanese in westernized environment and then to compare the results to native Japanese in Japan.

**Methods:**

Japanese adults in Pittsburgh who were registered at an urban primary care clinic were surveyed in terms of physical activity in daily life, exercise, and sedentary behavior. The results were compared to age- and gender-matched Japanese averages from the national data (Japanese National Health and Nutrition Survey, J-NHANS).

**Results:**

Of 97 identified for inclusion, all responded. Japanese in Pittsburgh did not engage physical activity in daily life as compared to J-NHANS results (p < .001 for both genders). Only 45.0% and 26.3% of Japanese men and women in Pittsburgh, respectively, reached the recommended level of exercise. The prevalence of regularly engaging moderate or vigorous level of exercise was significantly lower among Japanese in Pittsburgh than age- and gender-matched J-NHANS results. The prevalence of 2 hours or more per week of exercise at moderate or higher level among Japanese men and women in Pittsburgh were lower than J-NHANS results. Women in Pittsburgh showed significantly less sedentary time as compared to J-NHANS results, while men only showed significantly less sitting/lying time during weekend. We found no association between sedentary time (time in sitting/lying and TV/computer) and exercise time during weekday or weekend in the target population.

**Conclusions:**

Although Japanese in Pittsburgh showed lower prevalence of sedentary behavior, prevalence of regular physical activity and exercise were less than prevalence of native Japanese.

## Introduction

Noncommunicable chronic diseases (NCDs) such as cardiovascular disease, cancer, and diabetes are the leading causes of death globally [1]. The burden of these diseases is rising enormously [2]. To a large extent, NCDs are caused by behavioral risk factors in daily life. Physical inactivity is one of the major risk factors [3-5] and promoting physical activity (PA) and exercise to prevent NCDs has been called for [6,7].

The importance of PA has been shown extensively to prevent NCDs in various populations. Regular PA performed on most days of the week reduces the risk of premature death, coronary heart disease, diabetes, and colon cancer [7]. Regular PA also reduces blood pressure, promotes physiological well-being, and builds bones, muscles and joints [7]. Recently, evidence showed associations of sedentary behavior such as sitting, watching TV, and using computer with health independent of an individual’s PA [8,9]. Therefore, discouraging sedentary behavior is as important as promoting PA to prevent NCDs.

In addition, many studies on immigrants suggest that health status declines after staying in a different environment [10,11]. The declines are reported to be associated with lifestyle changes including physical inactivity to which they are exposed. Additionally studies investigating Japanese immigrants, one of the largest ethnic groups among Asian American ethnicities in USA, found significant increases in not only cardiovascular disease (CVD) risk factors but also its incidence and mortality rate when moving to western countries [12,13].

Despite the accumulating evidence on NCDs, two major knowledge gaps in the Japanese immigrants’ NCDs remain unfilled for the immigrant population. First, little is known about fundamental knowledge about type, frequency, and duration of PA and sedentary behavior in westernized environment. Due to the focus primarily on disease and disease-oriented risk factors, e.g., cancer, CVD, hypertension, dyslipidemia, and diabetes in the past studies, research on behavior that could lead effective intervention has not been tackled adequately [14]. Second, the association between PA and sedentary behavior has not been studied in this population, either.

The present study was to calculate the prevalence of PA in daily life and sedentary behavior among the Japanese in Pittsburgh, USA. Then, each of the prevalence was compared to age- and gender-matched Japanese national survey (the National Health and Nutrition Survey in Japan 2006, J-NHANS 2006 [15]) results. The study also determined the association between PA and sedentary behavior for the target population. Findings are of value in preparing for strategy to prevent NCDs in the population.

## Method

During September 2010 to February 2011, as reported elsewhere [16], all the Japanese patients who were age 18 years or older and registered (n = 97) at the Shadyside Family Health Center (FHC), University of Pittsburgh Medical Center, Pittsburgh, USA within the last 3 years as of August 1, 2010 were contacted by phone and requested to participate in the study. Japanese who registered our FHC as a primary care site but were less than 18 years old or had stayed in USA less than 3 months were excluded because of our specific objectives to assess adult Japanese in westernized environment.

At the initial contact through phone, each Japanese patient was asked to participate in the study after explaining the study goal, objective, process, and potential scientific impact. When the patient was interested in the study, we asked if the survey be answered at FHC or at home. Then, survey was answered in a preferred manner either at a private room in FHC or through mail.

Informed consent was obtained from all participants either by face-face (in case of answering at FHC) or through mail (in case of answering at home). The study was approved by the Institutional Review Board of University of Pittsburgh, Pittsburgh, USA. All the process was performed in Japanese. Materials used were also written in Japanese.

In this cross-sectional study, participants answered a self-administered 18 questions on PA in daily life, exercise, and sedentary behavior in the survey. PA is defined as any body movements more than resting in daily life. Exercise is defined as planned leisure activities to maintain and improve fitness. PA in daily life and exercise are further divided into 3 groups based on the metabolic equivalents (METS) intensity (light, moderate, and vigorous for <4, 4–6, and >6 METS, respectively) [17]. Sedentary behavior is defined as any behavior requiring less than 1.5 METS. The survey included two sedentary behavior categories (sitting/lying and TV/computer). The survey questions in the study were the same as the one used in J-NHANS 2006. J-NHANS is annual nationwide survey on health for Japanese. Based on Health Promotion Law in Japan, J-NHANS has been conducted to disclose physical condition of Japanese citizens, nutritional intake, and life condition. The participants were also asked their demographics. This study was concurrently conducted with other lifestyle assessment including nutrition, diet, stress, smoking, and alcohol intake. The study site (FHC) has been serving as a primary care center in the urban neighboring community setting. The site also has been playing the major role of caring for Japanese population in Pittsburgh due to our availability of multiple Japanese speaking physicians.

Demographic variables, e.g., age, weight, height, body mass index (BMI), and duration of being in USA, time for exercise, time for sedentary behavior, and health profile of the subjects were measured at the continuous level, while other variables were measured at the categorical level. Descriptive (frequency, mean, median, SD, range, etc.) statistics were used to describe the participant characteristics. The prevalence of PA in daily life and exercise characteristics was calculated and compared to age- and gender-matched results from the publicly available J-NHANS 2006 results. The participants were grouped into upper and lower half group with respect to their exercise time and sedentary time by using the median as a cutoff point in each of the behavior (exercise and sedentary) time among Japanese in Pittsburgh. To determine the correlation between exercise time and sedentary time for Japanese in Pittsburgh, Spearman’s rank correlation coefficients were calculated. Comparison between the characteristics and J-NHANS 2006 results (for each gender) was performed using either the chi-square test or student’s *t*-test. Assumptions of procedures were examined using Q-Q plots, box plots, and checking for inequality of variances. All statistical tests were two-tailed and reported P-values were unadjusted. The Predictive Analytics Software (PASW version 18.0, Somers, NY) was used for the analyses.

## Results

### Descriptive analyses

Table 1 displays the participant characteristics. Total of 97 Japanese patients (forty men and fifty-seven women) were identified from the FHC patient list and all agreed to participate in the study. All the 97 participants completed the survey. All the participants preferred to answer the survey by mail. Majority of the participants were married (95.0% and 98.2% for men and women, respectively) and had at least one child (77.5% and 82.5% for men and women, respectively). BMI for Japanese men and women in Pittsburgh was 23.8 kg/m^2^ and 21.3 kg/m^2^, respectively. Majority of men were employed whereas majority of women were homemakers. Around one tenth of men had history of hypertension and dyslipidemia, while only one woman had a history of hypertension. None of the participants had a history of diabetes or CVD.

**Table 1:** Demographic characteristics of study participants in Pittsburgh

	**Male (N = 40)**	**Female (N = 57)**	**Total (N = 97)**
Age, years	39.3 (6.5, [33.0])	36.6 (5.8, [27.0])	37.7 (6.2, [35.0])
Height, cm	172.3 (5.2, [29.0])	158.2 (5.5, [32.0])	164.1 (8.8, [41.0])
Weight, kg	70.7 (11.5, [60.0])	53.5 (8.7, [41.5])	60.6 (13.1, [69.5])
BMI, kg/m^2^	23.8 (3.4, [17.7])	21.3 (3.1, [15.7])	22.4 (3.4, [17.7])
Length of stay in USA, month	56.0 (55.3, [210.0])	46.7 (45.3, [186.0])	50.0 (49.5, [210.0])
Employee, N (%)	37 (92.5)	8 (14.0)	45 (46.4)
Marital status			
Single, N (%)	2 (5.0)	1 (1.8)	3 (3.1)
Married, N (%)	38 (95.0)	56 (98.2)	94 (96.9)
Have children, N (%)	31 (77.5)	47 (82.5)	78 (80.4)
Hypertension, N (%)	4 (10.0)	1 (1.8)	5 (5.2)
Dyslipidemia, N (%)	5 (12.5)	0	5 (5.2)
DM, N (%)	0	0	0
CHD, N (%)	0	0	0
Exercise engagement, N (%)			
Light	19 (47.5)	31 (54.4)	50 (51.5)
Moderate	16 (40.0)	22 (38.6)	38 (39.2)
Vigorous	19 (47.5)	15 (26.3)	34 (35.1)
Total┴	34 (85.0)	45 (78.9)	79 (81.4)
Exercise time, min/day*			
Light	10.5 (13.5)	9.1 (7.0)	9.6 (9.9)
Moderate	14.0 (12.3)	13.5 (14.0)	13.7 (13.1)
Vigorous	17.6 (15.9)	13.6 (8.1)	15.8 (13.0)
Total┴	21.6 (19.4)	16.9 (15.4)	18.9 (17.2)
Sedentary behavior			
Sitting/Lying, min/day			
Weekday	404.6 (266.3, [930])	274.4 (232.0, [960])	327.3 (253.4, [800])
Weekend	374.6 (246.1, [930])	212.7 (211.5, [780])	278.5 (238.7, [780])
TV/Computer time, min/day			
Weekday	313.1 (235.5, [660])	164.9 (155.2, [720])	225.1 (204.3, [780])
Weekend	235.4 (172.1, [690])	93.2 (96.7, [420])	151.0 (149.3, [720])

Data are mean (SD, [Range]) for continuous data and number (percentage) for categorical data when appropriate. ┴ Individual could have responded to more than 1 category resulting in frequency not adding to total.

*Among those who engage exercise at the level.

DM indicates diabetes mellitus; CHD, coronary heart disease.

### Physical activity in daily life, exercise, and sedentary assessment

Prevalence of PA in daily life, exercise and sedentary behavior among Japanese in Pittsburgh are displayed with corresponding J-NHANS results in the Tables 2 and 3.

**Table 2:** Comparison in Prevalence of Physical Activity and Exercise between Japanese Male and Female in Pittsburgh versus Age- and Gender-matched J-NHANS

**Characteristics**	**Male in Pitt**	**J-NHANS**	**Female in Pitt**	**J-NHANS**	**Total**	**J-NHANS**
		**(Male)**		**(Female)**	**In Pitt**	**(Total)**
PA in daily life	15.0*	51.8	12.3*	53.1	13.4	-
Walk 1 h or more/day	12.5*	63.3	12.3*	58.3	12.4*	60.6
Reaching recommended goal of exercise┴	45.0	-	26.3	-	34.0	-
Moderate PA in daily life						
No intention	27.5	19.4	10.5	12.0	17.5	15.4
Consider in future	30.0	29.1	43.9	28.0	38.1	28.5
Intend Not doing	32.5	18.6	28.1	28.1	29.9	23.7
Yes, but not regularly	7.5	8.6	7.0	9.3	7.2	9.0
Yes, every day	2.5	24.3	10.5	22.6	7.2	23.4
Vigorous PA in daily life						
No intention	20.0	30.6	22.8	26.1	21.6	28.2
Consider in future	25.0	32.3	50.9	35.7	40.2	34.1
Intend Not doing	30.0	15.4	17.5	21.2	22.7	18.5
Yes, but not regularly	17.5	9.5	5.3	7.1	10.3	8.2
Yes, every day	7.5	12.3	3.5	10.0	5.2	11.1
Highest level of exercise engaged						
None	15.0	82.5	19.3	82.5	17.5	82.5
Light	15.0	5.6	29.8	8.4	23.7	7.4
Moderate	22.5	7.4	24.6	6.5	23.7	6.8
Vigorous	47.5	4.6	26.3	2.6	35.1	3.3
Exercise Time > 2hours						
Light	15.0	18.8	8.8	18.4	11.3	18.6
Moderate	10.0	14.6	7.0	11.4	8.2	12.8
Vigorous	10.0	12.2	0.0	5.0	4.1	8.3

Pitt indicates Pittsburgh; J-NHANS, the National Health and Nutrition Survey in Japan; PA, physical activity. ┴ Moderate level exercise for 150 minutes a week or vigorous exercise for 60 minutes a week. *p < 0.001for comparison against J-NHANS.

**Table 3:** Comparison of Sedentary Behavior Rate between Japanese Male and Female versus Age- and Gender-adjusted J-NHANS

**Characteristics**	**Male in Pitt**	**J-NHANS**	**Female in Pitt**	**J-NHANS**
		**(Male)**		**(Female)**
Sitting/lying, weekday				
Longer half group├	50.0^n.s.^	50.8	45.6	73.0
Sitting/lying, weekend				
Longer half group┤	42.5*	60.6	33.3	78.3
TV/computer, Weekday				
Longer half group┬	52.5*	37.1	56.1*	73.0
TV/computer, Weekend				
Longer half group┬	42.5^n.s.^	54.7	28.9	81.2

├Sitting/lying time > 8 hours/day for male and > 6 hours/day for female.

┤Sitting/lying time > 6 hours/day for male and > 4 hours/day for female.

┬TV/computer time during weekday > 4 hours/day for male and >2 hours/day for female.

Pitt indicates Pittsburgh; J-NHANS, the National Health and Nutrition Survey in Japan.

*indicates p < 0.05; p < 0.001; n.s., not significant.

#### ***Characteristics of physical activity and exercise***

The prevalence of performing PA in daily life and walking for 1 hour or more per day were 15.0% and 12.5% among Japanese men in Pittsburgh and 12.3% and 12.3% in Japanese women in Pittsburgh. The prevalence of performing PA in daily life and walking for 1 hour or more per day were significantly lower among Japanese in Pittsburgh than J-NHANS results for both genders as shown in Table 2. The prevalence of regularly engaging moderate or vigorous exercise were 2.5% and 7.5% among Japanese men and 10.5% and 3.5% among Japanese women in Pittsburgh. The prevalence of performing exercise for 2 hour or more per week in all of the 3 intensities (light, moderate, and vigorous) were also lower for both genders in Pittsburgh as compared to J-NHANS results, although the difference were not statistically significant. Only 45.0% of Japanese men and 26.3% of Japanese women in Pittsburgh met the recommended exercise criteria published in the guideline [18].

#### ***Characteristics of sedentary behavior***

The prevalence of median-split longer half group for sitting/lying and TV/computer during weekday and weekend among Japanese men in Pittsburgh were shown in Table 3. Only the prevalence of longer half group for sitting/lying during weekend was significantly lower among Japanese men in Pittsburgh than J-NHANS results. Among Japanese women in Pittsburgh, the prevalence of longer half group for sitting/lying and TV/computer during weekday and weekend were also shown in Table 3. All of the prevalence of the sedentary behaviors among Japanese women in Pittsburgh were significantly shorter than those of J-NHANS results.

#### ***Correlation between exercise and sedentary behavior***

Total time in exercise was not associated with time of sitting/lying and time of TV/computer during weekday (p = 0.94, and 0.35) and weekend (p = 0.91 and 0.24) among Japanese men in Pittsburgh. Similarly, among Japanese women in Pittsburgh, total time in exercise was not associated with time of sitting/lying and time of TV/computer during weekday (p = 0.26 and 0.22) and weekend (p = 0.72 and 0.40).

## Discussion

The prevalence of both regular PA in daily life and exercise among Japanese in Pittsburgh were less than age- and gender-matched J-NHANS results. With respect to sedentary behavior, this study found Japanese men in Pittsburgh exhibited significantly shorter in only one sedentary behavior category (sitting/lying time during weekend) as compared to the corresponding J-NHANS results, whereas Japanese women in Pittsburgh spent significantly less time in all sedentary behavior categories as compared to J-NHANS results. In addition, no correlation between exercise time and sedentary time for either genders was found.

The most striking findings in PA in daily life and exercise were that the prevalence of PA in daily life and percentage of meeting the recommended criteria of exercise were only 15.0 and 45.0% among Japanese men in Pittsburgh and 12.3 and 26.3% among Japanese women in Pittsburgh, respectively. The prevalence of meeting the recommended exercise criteria was low because Japanese in Pittsburgh engaged non-regular exercise and short time for moderate and vigorous exercise, although the prevalence of performing any intensity of exercise was not low in the target population. The study of the same participants showed that majority of them increased weight and decreased in exercise after the entry to USA [16]. Our findings in this study may be related to the sign of newly adopted behavior in the westernized environment. Based on our results, the effort concerning exercise promotion should be made in establishing regularity of exercise and increasing time to engage exercise in addition to increasing PA in daily life as the preventive strategy for NCDs.

We also found Japanese men in Pittsburgh only showed significantly less sitting/lying time during weekend as compared to J-NHANS results. The time for sitting/lying and TV/computer are shown to be associated with unhealthy outcome such as obesity, dyslipidemia, hypertension and type 2 diabetes [19-23]. Therefore, health promotion intervention to the sedentary behavior may be beneficial for Japanese men in Pittsburgh. On the other hands, higher percentage of Japanese women in Pittsburgh showed less sedentary behavior in comparison to J-NHANS results. We need to interpret these results carefully when considering extra intervention to lessen the time for sedentary behavior lead benefit of preventing NCDs for women in Pittsburgh whose prevalence of PA in daily life and exercise was low. Although sitting time [22] and television time [23] are shown to be the metabolic risk of cardiovascular disease independent of PA and exercise, appropriate level of sedentary behavior across the different intensity of PA and exercise is unclear. Additionally there currently exists no clear goal of sedentary behavior in the national health promotion campaign (Japan National Health Promotion in the 21st Century [24]). Therefore, further investigation is needed to conclude if intervention to lessen the time for sedentary behavior is required for women.

With respect to the relation between exercise and sedentary behavior, we found no correlation between these behaviors among both men and women moving to westernized environment. Rouse et al. reported that these two behaviors in university students were uncorrelated [25]. The accumulating evidence with which our findings were consistent suggests that exercise promotion by reducing sedentary behavior may have limited success in this population.

There are several strengths in this study. First, the response rate was very high (100.0%). In addition to the high response rate, all the Japanese at FHC where care were provided in the community primary care setting agreed to participate in the study. This means the study participants likely represented the Japanese community in Pittsburgh who seek standard care in primary care settings. Second, the present paper provided not only descriptive summary on PA in daily life, exercise and sedentary behavior but also meaningful analyses to suggest strategies to prevent NCDs by comparing our results and J-NHANS data. J-NHANS data allowed age- and gender-matched comparisons in the same ethnicity. Our study has strength from ethnic perspectives because studies specific to each of the Asian population are sparse. Asian Americans, who are the fastest growing among all major ethnic or racial groups in USA, are so diverse in health and diseases so grouping into a single group conceals the difference among them [26]. This study is one of a few studies specifically targeting the ethnicity.

This study has some limitations. This is a cross-sectional study. Although we used the same questionnaire in the survey as the J-NHANS used, bias such as reporting and recall bias cannot be completely eliminated due to the self-administration. Additionally, although existing data allow us to compare our results with the age- and gender-matched national averages, we did not perform any multivariable adjustments including cardiovascular risk factors.

## Conclusion

This paper provides evidence of less prevalence of regular PA in daily life and exercise among Japanese in Pittsburgh than native Japanese. Behavior intervention to increase both regular PA in daily life and exercise is suggested without increasing their sedentary behavior to prevent NCDs.

## Competing interests

The authors declare that they have no competing interests.

## Authors’ contributions

NH designed the study, collected the data, analyzed the data, and drafted the manuscript. TT made substantial contribution to the study design, the data acquisition, and revising the manuscript. FD made substantial contribution to the design, data analysis, drafting the manuscript, and critically revising the manuscript. All authors read and approved the final manuscript.
